# Supplementary material for: Microstructural and functional plasticity following repeated brain stimulation during cognitive training in older adults
Source: Nat Commun. 2023 Jun 2;14:3184. doi: 10.1038/s41467-023-38910-x (PMC10238397; doi:10.1038/s41467-023-38910-x)
Supplement: Supplementary file 1 — Supplementary Information [file 41467_2023_38910_MOESM1_ESM.pdf]

Supplementary Information

Microstructural and functional plasticity following repeated brain stimulation during cognitive training in older adults

Antonenko et al. Nature Communications 2023

Supplementary Methods and Results

Adverse events and blinding

Safety outcomes are reported separately as incidences (n, incidence rate with 95%-CI, based on poisson regression models) in total and by intervention group. Ten adverse events were reported by seven participants in the target (active stimulation) group and 14 adverse events were reported by eight participants in the control (sham tDCS) intervention group (Supplementary Table 1). No serious adverse events were reported and no participant terminated participation due to occurrence of adverse events.

Supplementary Table 1. **Self-reported incidence of adverse events (at least moderate symptoms) by group during intervention.**

	Total N=48	Target (active stimulation) intervention group n = 22	Control (sham) intervention group n = 26	Incidence rate ratio for group differences (95 % CI)
Observation time in days, mean (SD)	9.0 (0.1)	9.0 (0.2)	9.0 (0)	
Total number of adverse events	24/ 5.6 (3.6-8.1)	10/ 5.1 (2.5-8.9)	14/ 6.0 (3.4-9.7)	0.8 (0.4-1.9)
Itching	7/ 1.6 (0.7-3.1)	4/ 2 (0.6-4.7)	3/ 1.3 (0.3-3.3)	1.6 (0.3-8.0)
Pain	3/ 0.7 (0.2-1.8)	0/ 0	3/ 1.3 (0.3-3.3)	-
Burning	7/ 1.6 (0.7-3.1)	4/ 2 (0.6-4.7)	3/ 1.3 (0.3-3.3)	1.6 (0.3-8.0)
Warmth/heat	0 / -	0/ -	0 / -	-
Metallic/iron taste	1/ 0.2 (0-1)	0/ -	1/ 0.4 (0-1.9)	-
Fatigue	2/ 0.5 (0.1-1.4)	1/ 0.5 (0-2.2)	1/ 0.4 (0-1.9)	1.2 (0-30)
Other	4/ 0.9 (0.3-2.2)	1/ 0.5 (0-2.2)	3/ 1.3 (0.3-3.3)	0.4 (0-3)

Note: Reported values are absolute frequency of the respective AEs / incidence rate per 100 patient days (95 % CI). Source data are provided as a Source Data file.

At the end of all training sessions, participants were asked to guess to which treatment group they were assigned at randomization, see Supplementary Table 2 for an overview of the answers. We computed the James Blinding Index (BI) where a value of 0.5 (ranging from 0: lack of blinding with all answers correct, to 1: lack of blinding with all answers incorrect; 0.5 means half of the answers are correct, half incorrect) represents random guessing in a randomized, clinical study [1, 2]. The estimate was 0.67 (95%-CI: 0.55 to 0.80), indicating blinding success.

Supplementary Table 2. **Number of participants by group assignment and guess.**

Assignment	Response			
	Target	Control	DK	Total
Target	14	3	5	22
Control	10	3	13	26
Total	24	8	16	48

Note: DK denotes "Don't know". Source data are provided as a Source Data file.

Tract-Based Spatial Statistics (TBSS)

Voxel-wise statistical analysis of the FA data was carried out using TBSS (Tract-Based Spatial Statistics) [3], part of FSL [4]. Briefly, FSL's nonlinear image registration algorithm was used to align all subjects' FA images to the FMRIB58_FA template in Montreal Neurological Institute (MNI) standard space. A mean skeleton was created which represents the centres of all tracts common to the group. Afterwards each subjects' aligned FA data was projected onto this skeleton (using a lower threshold of 0.2 to include only white matter and reduce the likelihood of partial voluming) and the resulting data was fed into whole-brain voxel-wise cross-subject statistics. The "randomize" algorithm with 5000 permutations was applied, with a cluster significance level of $p < 0.05$ using threshold-free cluster enhancement (TFCE) to control for multiple

comparisons over space[5] (and a minimum cluster size threshold 'k' of 20 voxels). Paired within-subject Post-Pre differences were computed and subjected to a standard general linear model design (similar to our whole-brain voxel-wise statistical design for the resting-state data in CONN). With this approach, a two-sample t-test was performed to test whether the Post-Pre difference differed between groups (specifically testing the contrast of a relative FA increase in the anodal compared to the sham group), adjusted for age and sex.

A significant relative FA increase in anodal compared to sham group was found in left and right lateral prefrontal, medial prefrontal and parietal regions (permutation test, $p < 0.05$, TFCE-corrected, Supplementary Table 3 and Supplementary Fig. 1). Cluster sizes and center of gravity cluster MNI coordinates were extracted and regions were labeled with references to John Hopkin University (JHU) white matter (WM) atlas [6]. Atlas labels mostly corresponded to fiber systems overlapping with the canonical probabilistic pathway (Supplementary Fig. 2).

Supplementary Table 3. **Clusters of relative FA increase in the anodal compared to sham group.**

Associated cortical regions	Cluster size (mm^3)	Minimum p (TFCE-corrected)	MNI coordinates x	y	z	Hemi
Cingulum/CC	155	0.002	-7.4	5.9	31.1	LH
	21	0.022	8.8	11.1	31.4	RH
SLF	58	0.028	-30.6	-15.2	50.9	LH
	36	0.018	-6.6	-10.9	56.1	LH
	32	0.012	-37.2	-7.7	46.9	LH
	25	0.032	-36.3	14.6	40.1	LH
	25	0.017	-43.0	-56.3	34.3	LH
	24	0.035	-41.1	-4.3	41.0	LH
	20	0.027	-44.9	3.6	16.6	LH
	38	0.036	27.1	3.1	27.2	RH
	31	0.027	49.8	3.4	29.3	RH
	28	0.023	42.7	-3.8	42.9	RH
	27	0.021	31.3	3.4	35.0	RH
	21	0.029	38.1	1.4	25.1	RH
ATR	24	0.012	8.2	-31.4	-10.7	RH
Cerebellum	28	0.018	-11.4	-64.9	-32.2	LH

Note: MNI coordinates (in mm) are given for the center of gravity of the clusters. Neuroanatomical regions were labeled with reference to the John Hopkins University (JHU) atlas. CC, corpus callosum. SLF, superior longitudinal fasciculus. ATR, anterior thalamic radiation. Hemi, hemisphere. LH, left hemisphere. RH, right hemisphere. Permutation testing, two-sided p<0.05 and multiple comparison correction using threshold-free cluster enhancement.

Supplementary Information

Microstructural and functional plasticity following repeated brain stimulation during cognitive training in older adults

Antonenko et al. Nature Communications 2023

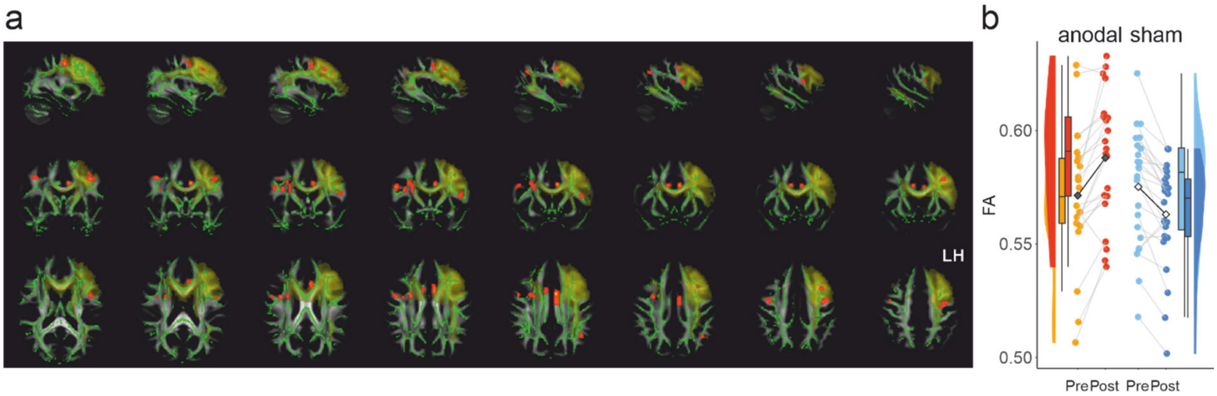

Supplementary Figure 1. **Tract-based spatial statistics (TBSS) analysis. a** Sagittal (top row), coronal (middle row) and axial (bottom row) view. Relative increase of fractional anisotropy (FA) in the anodal compared to sham group. The effects were estimated by a whole-brain comparison of anodal and sham group by means of voxel-wise general linear model (GLM, group x time interaction). Red regions (thickened for better visibility) represent tracts with increased FA (permutation test, $p<0.05$, TFCE-corrected, $k \geq 20$). Results are overlaid on the mean FA skeleton (green) and FSL_HCP1065-FA template (grey). The canonical pathway derived from probabilistic tractography analysis is superimposed (yellow) to illustrate overlap with significant TBSS clusters. **b** Means (black diamonds for andodal and white diamonds for sham) and individual data points (single circles in orange/red for anodal and lightblue/ darkblue for sham). Box plots indicate median (middle line), 25th, 75th (box), and 5th and 95th percentile (whiskers). *n* = 46 independent participants. LH, left hemisphere. Source data are provided as a Source Data file.

Tracts constrained by underlying anatomy (TRACULA)

We explored our tractography outcome (i.e., the canonical pathway) more closely and overlaid it with atlas labels of the John's Hopkins University (JHU) white matter (WM) atlas (Supplementary Fig. 2). The reconstructed tracts connect bilateral prefrontal areas, thus overlapping with the prefrontal part of the body of the corpus callosum (CC); and left lateral prefrontal with left parietal areas, thus overlapping with the superior frontal fasciculus (SLF). We then reconstructed these two specific fiber systems (CC, SLF; and extracted the average FA values) for all individuals and timepoints, see below for details.

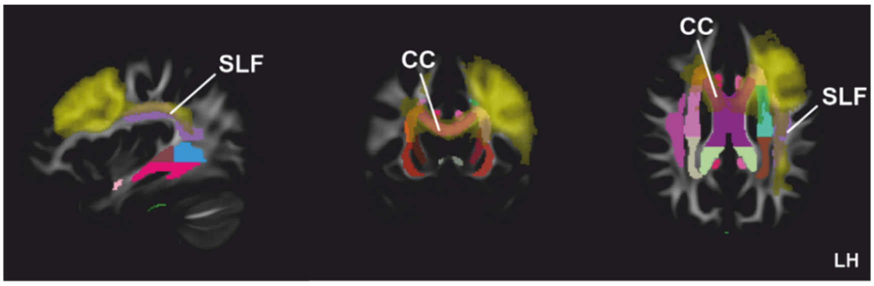

Supplementary Figure 2. **Overlay of the canonical tract derived from the probabilistic tractography (yellow) and the JHU WM atlas labels (multicolored).** SLF, superior longitudinal fasciculus. CC, corpus callosum. LH, left hemisphere.

To reconstruct specific fiber systems, automated global tractography with anatomical priors was carried out on the preprocessed FA images (processed with the longitudinal pipeline, see Methods Section) using the tracts constrained by underlying anatomy (TRACULA) tool included in FreeSurfer version 7 [7]. Pathways were estimated based on the training subjects' atlas data combined with the individual segmentation data. Analyses were focused on fibers overlapping with our probabilistic pathway, i.e. connections of the left prefrontal/middle frontal areas (Supplementary Fig. 2): Corpus callosum (CC), prefrontal section of the body (defined based on its cortical terminations in the rostral subdivision of the superior frontal label or in the rostral middle frontal label) and left superior longitudinal fasciculus (SLF), 2nd branch as defined by

anatomical literature (with inclusion ROI in the caudal part of the middle frontal gyrus and in the inferior parietal lobe, and mid-sagittal and temporal exclusion ROI) [7]. A-posteriori probability distributions were estimated consisting of a likelihood term (estimated from the ball-and-stick model of diffusion) and a term including the estimated pathway priors. FA values were calculated by averaging the individual voxel values along the CC and the SLF for each participant and timepoint.

FA values for each participant and timepoint are displayed in Supplementary Fig. 3. FA values were entered into linear model analyses with values post intervention as dependent variables and group as between-subjects factor (including pre intervention values, age, and sex as covariates). FA values in the CC were higher in the anodal compared to the sham group (main effects $t_{40} = -1.96$, $p = 0.058$, partial $\eta^2 = 0.09$) and an interaction of initial FA values by group was found ($t_{40} = 2.01$, $p = 0.051$, partial $\eta^2 = 0.09$). Thus, beneficial stimulation effects were larger for individuals with higher FA at baseline (e.g., for low baseline values at 25th percentile (0.52), anodal: 0.52 [0.51, 0.53], sham: 0.52 [0.51, 0.53], p = 0.530; for high baseline values at 75th percentile (0.57), anodal: 0.57 [0.56, 0.58], sham: 0.56 [0.55, 0.57], p = 0.089). FA in the SLF did not change through the intervention ($t_{41} = 0.02$, $p = 0.984$, partial $\eta^2 = 9.9e{-}06$; model-derived estimated means [CI]: 0.42 [0.41, 0.42] for anodal and 0.42 [0.41, 0.42] for sham group). In sum, we found increased FA values in the CC (prefrontal section of the body) in anodal compared to sham for individuals with higher baseline FA while no difference was observed for the left SLF.

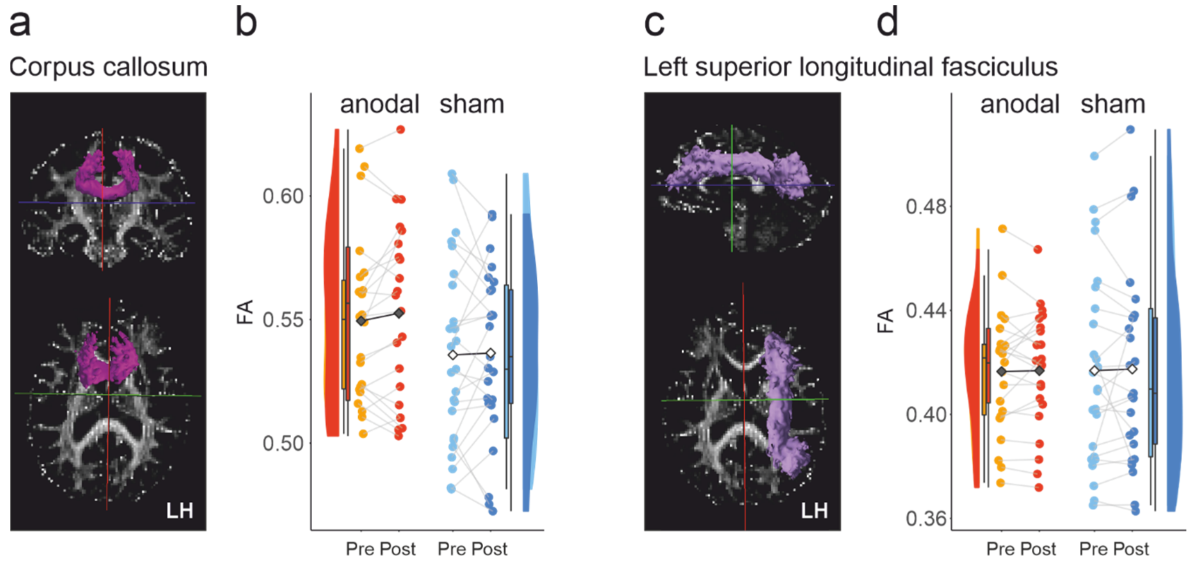

Supplementary Figure 3. **TRACULA analysis. a+c** Two specific fiber tracts of interest (Corpus callosum, CC, and left superior longitudinal fasciculus, SLF) reconstructed using FreeSurfer's TRACULA (v7). Sample tracts of one representative participant, overlaid on the individual fractional anisotropy (FA) image, are shown. **b+d** Means (black diamonds for andodal and white diamonds for sham) and individual data points (single circles in orange/red for anodal and lightblue/ darkblue for sham). Box plots indicate median (middle line), 25th, 75th (box), and 5th and 95th percentile (whiskers). *n* = 46 independent participants. FA along the CC was increased after the intervention in anodal compared to sham group for those individuals with initially higher FA in the tract. FA along the SLF did not change through the intervention. LH, left hemisphere. Source data are provided as a Source Data file.

Association approach

We also computed possible linear models for the two dependent variables (performance change in N-back and performance change in LU training task). These models included all three levels of neural modulation which were studied (FA change in the tract, MD change in the target, and FC change between the target and the significant right-hemispheric cluster) as independent variables (Supplementary Table 4). For N-back change, FA difference from before to after the intervention still showed a positive relationship, despite

inclusion of other the variables (t_{40}=2.57, p=0.009). For LU change, none of the neural markers showed a relationship (the association with FC change becoming non-significant, t_{41}=1.38, p=0.174, most probably due to our observation from the scatterplots that it was only present in the anodal group). As the link between the dependent variables is not evident from these models, we computed an additional model including MD change as the independent variable and FA change and FC change as covariates. This model showed a less pronounced relationship of MD and FC change (t_{42}=-1.78, p=0.082) than the unadjusted bivariate correlation.

Supplementary Table 4. **Linear regression analyses.**

	B	SE	t	*p*
N-back change				
Intercept	2.40	0.94	2.57	0.014
FA change	183.0	67.29	2.72	0.009
MD change	0.12	9.49	0.01	0.990
FC change	2.50	5.83	0.43	0.671
LU change				
Intercept	4.40	0.49	9.10	2.24e-11
FA change	15.60	35.00	0.45	0.658
MD change	-1.74	4.87	-0.36	0.723
FC change	4.20	3.04	1.38	0.174
MD change				
Intercept	0.022	0.01	1.48	0.147
FA change	-0.022	1.11	-0.02	0.984
FC change	-0.17	0.09	-1.79	0.082

Note: Linear models, two-sided, no correction for multiple comparisons. Source data are provided as a Source Data file.

Microstructural and functional plasticity following repeated brain stimulation during cognitive training in older adults

Antonenko et al. Nature Communications 2023

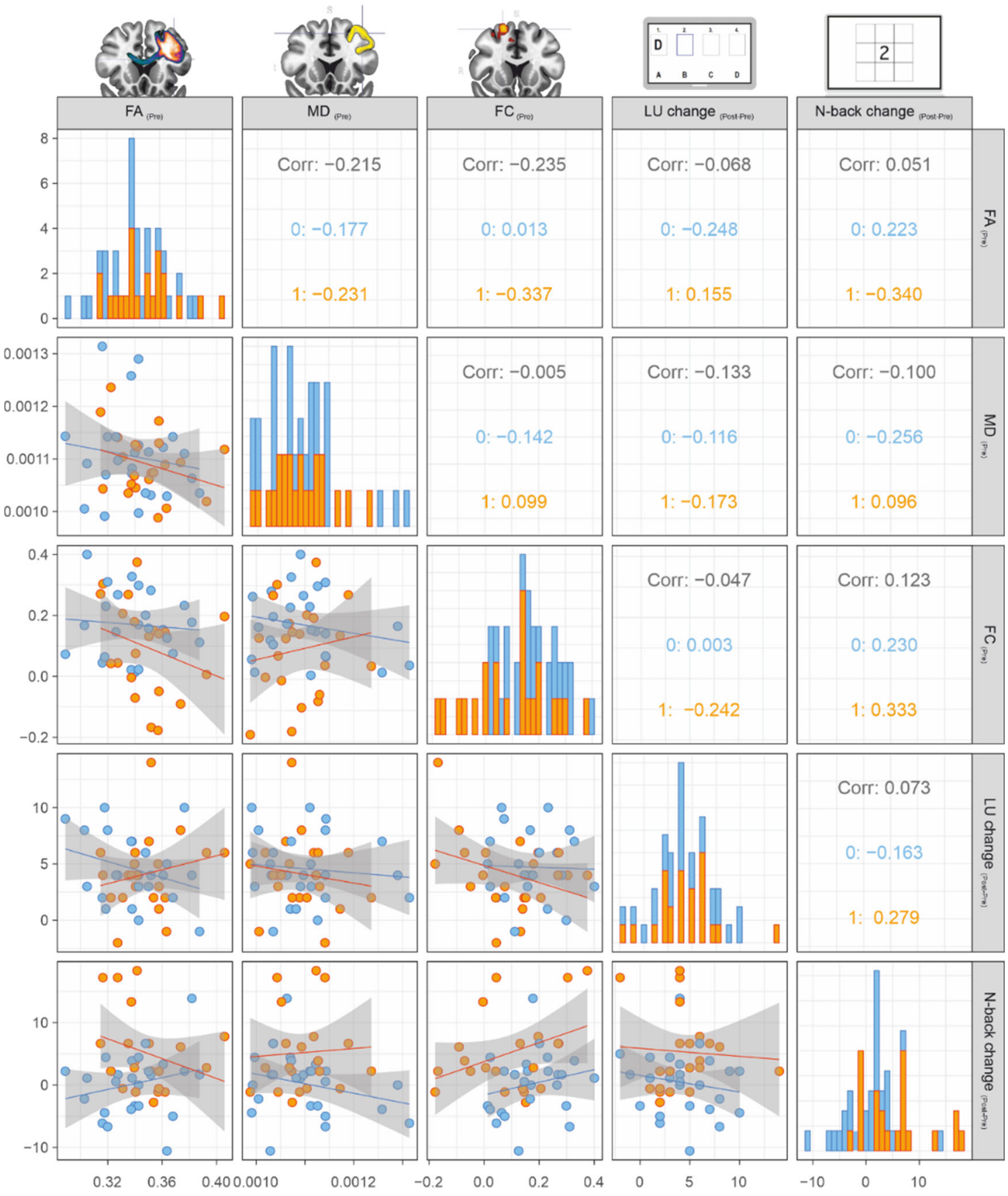

Supplementary Figure 4. **Scatterplots for correlations between baseline FA, MD, and FC (values at Pre) with individual performance gain (LU and N-back change).** Brain images were created with MRIcroGL (https://www.nitrc.org/projects/mricrogl). FA, fractional anisotropy. MD, mean diffusivity. FC, functional connectivity. LU, letter updating. Blue bars/points/0: sham group. Orange bars/points/1: anodal tDCS group. Shaded areas represent the 95% confidence interval. Source data are provided as a Source Data file.

Supplementary Information

Microstructural and functional plasticity following repeated brain stimulation during cognitive training in older adults

Antonenko et al. Nature Communications 2023

Simulation of electric field

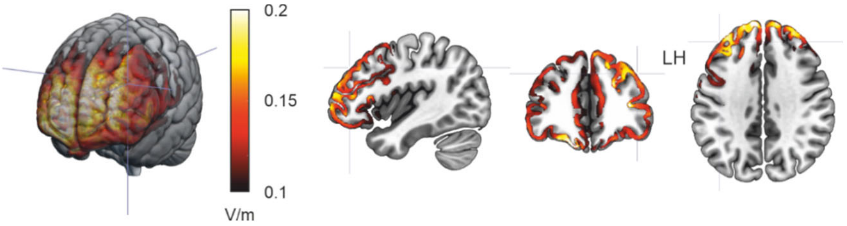

Supplementary Figure 5. **Electric field distribution.** 3D, sagittal, coronal, and axial views of the field simulated for the applied stimulation protocol on an MNI brain using SimNibs [8]; anode centered over the left dorsolateral prefrontal cortex (F3, 5-cm diameter, 1 mA) and cathode centered over the contralateral supraorbital region (Fp2, 5-cm diameter, 1 mA). Brain images were created with MRIcroGL (https://www.nitrc.org/projects/mricrogl). Field magnitude below the anodal electrode: ~0.15 V/m. LH, left hemisphere.

Supplementary References

1. Arroyo-Fernandez R, Avendano-Coy J, Velasco-Velasco R, Palomo-Carrion R, Bravo-Esteban E, Ferri-Morales A. A New Approach to Assess Blinding for Transcranial Direct Current Stimulation Treatment in Patients with Fibromyalgia. A Randomized Clinical Trial. *Brain Sci* **11**, 1335 (2021).
2. Bang H, Ni L, Davis CE. Assessment of blinding in clinical trials. *Control Clin Trials* **25**, 143-156 (2004).
3. Smith SM*, et al.* Tract-based spatial statistics: voxelwise analysis of multi-subject diffusion data. *NeuroImage* **31**, 1487-1505 (2006).
4. Smith SM*, et al.* Advances in functional and structural MR image analysis and implementation as FSL. *NeuroImage* **23 Suppl 1**, S208-219 (2004).
5. Smith SM, Nichols TE. Threshold-free cluster enhancement: addressing problems of smoothing, threshold dependence and localisation in cluster inference. *NeuroImage* **44**, 83-98 (2009).
6. Mori S*, et al.* Stereotaxic white matter atlas based on diffusion tensor imaging in an ICBM template. *NeuroImage* **40**, 570-582 (2008).
7. Maffei C*, et al.* Using diffusion MRI data acquired with ultra-high gradient strength to improve tractography in routine-quality data. *NeuroImage* **245**, 118706 (2021).
8. Saturnino GB, Puonti O, Nielsen JD, Antonenko D, Madsen KH, Thielscher A. SimNIBS 2.1: A Comprehensive Pipeline for Individualized Electric Field Modelling for Transcranial Brain Stimulation. In: *Brain and Human Body Modeling: Computational Human Modeling at EMBC 2018* (eds Makarov S, Horner M, Noetscher G). Springer Copyright 2019, The Author(s). (2019).
