# Supplementary material for: Microstructural and functional plasticity following repeated brain stimulation during cognitive training in older adults
Source: Nat Commun. 2023 Jun 2;14:3184. doi: 10.1038/s41467-023-38910-x (PMC10238397; doi:10.1038/s41467-023-38910-x)
Supplement: Supplementary file 3 — Reporting Summary [file 41467_2023_38910_MOESM3_ESM.pdf]

nature portfolio

Corresponding author(s): Daria Antonenko

Last updated by author(s): April 25, 2023

Reporting Summary

Nature Portfolio wishes to improve the reproducibility of the work that we publish. This form provides structure for consistency and transparency in reporting. For further information on Nature Portfolio policies, see our Editorial Policies and the Editorial Policy Checklist.

Statistics

For all statistical analyses, confirm that the following items are present in the figure legend, table legend, main text, or Methods section.

n/a	Confirmed	
☐	☒	The exact sample size (*n*) for each experimental group/condition, given as a discrete number and unit of measurement
☐	☒	A statement on whether measurements were taken from distinct samples or whether the same sample was measured repeatedly
☐	☒	The statistical test(s) used AND whether they are one- or two-sided<br>*Only common tests should be described solely by name; describe more complex techniques in the Methods section.*
☒	☐	A description of all covariates tested
☐	☒	A description of any assumptions or corrections, such as tests of normality and adjustment for multiple comparisons
☐	☒	A full description of the statistical parameters including central tendency (e.g. means) or other basic estimates (e.g. regression coefficient) AND variation (e.g. standard deviation) or associated estimates of uncertainty (e.g. confidence intervals)
☐	☒	For null hypothesis testing, the test statistic (e.g. *F*, *t*, *r*) with confidence intervals, effect sizes, degrees of freedom and *P* value noted<br>*Give P values as exact values whenever suitable.*
☒	☐	For Bayesian analysis, information on the choice of priors and Markov chain Monte Carlo settings
☒	☐	For hierarchical and complex designs, identification of the appropriate level for tests and full reporting of outcomes
☒	☐	Estimates of effect sizes (e.g. Cohen's *d*, Pearson's *r*), indicating how they were calculated

Our web collection on statistics for biologists contains articles on many of the points above.

Software and code

Policy information about availability of computer code

Data collection	MRI data acquisition was performed using the corresponding data acquisition software of the manufacturer (Siemens Verio 3T; SIEMENS MAGNETOM Verio syngo MR B17); Tasks were programmed with E-prime 3.0 and in-house software programmed using Unity, C++ and Visual Basics .NET 15.
Data analysis	Sample calculation was done with G*Power 3.1. Data analysis was performed with Matlab v2019a (The MathWorks, Inc. Natick, MA, USA) using SPM 12 and CONN toolbox v21 (www.nitrc.org/projects/conn) for resting-state fMRI; Freesurfer v6 (https://surfer.nmr.mgh.harvard.edu) and FSL with BEDPOSTX v6 for DTI/T1 image processing.

For manuscripts utilizing custom algorithms or software that are central to the research but not yet described in published literature, software must be made available to editors and reviewers. We strongly encourage code deposition in a community repository (e.g. GitHub). See the Nature Portfolio guidelines for submitting code & software for further information.

Data

Policy information about availability of data

All manuscripts must include a data availability statement. This statement should provide the following information, where applicable:

- Accession codes, unique identifiers, or web links for publicly available datasets
- A description of any restrictions on data availability
- For clinical datasets or third party data, please ensure that the statement adheres to our policy

The processed data of this study are available upon reasonable request from the corresponding author. The raw data are not publicly available due to potential identifying information that could compromise participant privacy. Source data are provided with the paper, where the relevant data from each figure or table is represented by a single sheet within the source data file (https://github.com/annaelisabethfromm/NCOM_Antonenko_2023).

Furthermore, we used the following data bases:
Freesurfer: Desikan-Killiany (parcellation) Atlas (https://surfer.nmr.mgh.harvard.edu/fswiki/CorticalParcellation)
CONN: Harvard-Oxford Atlas (https://web.conn-toolbox.org/resources/conn-in-pictures)
TRACULA : White-matter tract atlas (https://dmri.mgh.harvard.edu/tract-atlas/)

Research involving human participants, their data, or biological material

Policy information about studies with human participants or human data. See also policy information about sex, gender (identity/presentation), and sexual orientation and race, ethnicity and racism.

Reporting on sex and gender	Research findings apply to both sexes, because we included both female and male participants in the study (31 female, 17 male). All analyses reported in the paper are controlled for the effects of age and sex (self-reported). Gender issues were not considered in the data analysis.
Reporting on race, ethnicity, or other socially relevant groupings	Not applicable.
Population characteristics	48 nondemented/healthy older adults (31 female) aged 69.8/3.9 years (mean/SD) without history of neurological or severe psychiatric disease; education: 15.5/2.2 years (mean/SD), CERAD total score: 88.4 (4.8) (mean/SD; max. 100).
Recruitment	Participants were recruited from local newspapers distribution of flyers in local senior citizen clubs. We are not aware of any self-selection bias, because participants were randomized equally by a researcher unaware of group assignment into the stimulation groups using using block wise randomization (https://CRAN.R-project.org/package=blockrand). For more details see "Randomization".
Ethics oversight	Ethics committee of the University Medicine Greifswald

Note that full information on the approval of the study protocol must also be provided in the manuscript.

Field-specific reporting

Please select the one below that is the best fit for your research. If you are not sure, read the appropriate sections before making your selection.

☒ Life sciences ☐ Behavioural & social sciences ☐ Ecological, evolutionary & environmental sciences

For a reference copy of the document with all sections, see nature.com/documents/nr-reporting-summary-flat.pdf

Life sciences study design

All studies must disclose on these points even when the disclosure is negative.

Sample size	An a priori power analysis was performed to determine sample size for the behavioral primary outcome as defined in the study protocol paper. Based on recent studies in the field using multi-session application of anodal tDCS during cognitive training compared to training with sham tDCS, we estimated an effect size of 0.85. To demonstrate an effect in the primary outcome, 46 participants (23 per group) need to be included in the analysis with an independent t-test using a two-sided significance level of 0.05 and a power of 80%. This conservative approach using a t-test was chosen, even though we intend to analyze the primary outcome conducting analysis of covariance (ANCOVA) models . Assuming a drop-out rate of about 20% due to a high number of planned visits and considerably high demands put upon participants (e.g., performing challenging memory tasks and attending three sessions of 45 min MRI scans), 28 participants should be included in each tDCS group. Sample size estimation was conducted using G*Power 3.1 (https://www.psychologie.hhu.de/arbeitsgruppen/allgemeinepsychologie-und-arbeitspsychologie/gpower). MRI was a secondary outcome for which no sample size calculation was performed. Only participants which had MRI scans at pre and post assessment were included. Taking into account previous literature, were were convincedthat our sample size would be adequate to achieve the aims of our study.
Data exclusions	Resting-state fMRI: Exclusion of n=1 from sham groupdue to exessive motion during the functional scan (exceeding 3 SD of the sample mean number of outlier scans (rendering data quality too low for appropriate analyses); DTI data: Exclusion of n=2 (one from anodal in post, one

from sham in pre) in tractography due to missing (not acquired) DTI data.

Replication

The experiment was performed by the same researchers over all sessions. A number of control analyses with a range of manipulations were implemented to validate the results. Statistical tests were performed by multiple designs and models and all fundamental results were replicated.

Randomization

Fifty-six eligible participants were randomly allocated to target and control intervention group with a 1:1 ratio, using age and initial performance in the letter updating task as strata. First, all participants that successfully completed telephone screening and baseline assessments were divided into four groups by median split: (1) age <= median and LU performance <= median, (2) age <= median and LU performance > median, (3) age > median and LU performance <= median, and (4) age > median and LU performance > median. Second, equal numbers of participants of each group were randomly assigned to anodal and sham tDCS group, respectively, using the blockrand package in R software (https://www.r-project.org).

Blinding

tDCS administeration during task performance was single-blind, however, investigators were blind to group allocation during MRI data acquisition and analysis.

Reporting for specific materials, systems and methods

We require information from authors about some types of materials, experimental systems and methods used in many studies. Here, indicate whether each material, system or method listed is relevant to your study. If you are not sure if a list item applies to your research, read the appropriate section before selecting a response.

Materials & experimental systems

n/a	Involved in the study
☒	☐ Antibodies
☒	☐ Eukaryotic cell lines
☒	☐ Palaeontology and archaeology
☒	☐ Animals and other organisms
☒	☐ Clinical data
☒	☐ Dual use research of concern
☒	☐ Plants

Methods

n/a	Involved in the study
☒	☐ ChIP-seq
☒	☐ Flow cytometry
☐	☒ MRI-based neuroimaging

Magnetic resonance imaging

Experimental design

Design type

Eyes-closed resting-state functional MRI (plus DTI and T1 anatomical scan)

Design specifications

6-min acquisition (172 volumes)

Behavioral performance measures

N/A. No task was administered during MRI acquisition.

Acquisition

Imaging type(s)

Diffusion, structural, functional

Field strength

3T

Sequence & imaging parameters

Resting-state fMRI scans were acquired using an echo-planar-imaging sequence (3 x 3 x 3 mm^3 voxel size, repetition time (TR) = 2000 ms, echo time (TE) = 30 ms, flip angle = 90 °, 34 slices, descending acquisition, field of view 192 x 192 mm^2, 176 volumes, TA = 6.00 min). Participants were instructed to keep their eyes closed, to not think of anything in particular, and to try not to fall asleep. High-resolution anatomical images were acquired using a three-dimensional T1-weighted magnetization prepared rapid gradient echo imaging (1 mm^3 isotropic voxel, TR = 2300 ms, TE = 2.96 ms, inversion time = 900 ms, flip angle = 9°, 256 x 240 x 192 mm^3 matrix).

Area of acquisition

Whole-brain

Diffusion MRI ☒ Used ☐ Not used

Parameters

Further, a diffusion-weighted spin-echo echo-planar imaging sequence was acquired (1.8 x 1.8 x 2.0 mm^3 voxel size, TR = 11100 ms, TE = 107 ms, 70 slices, 64 directions (b = 1000 s/mm^2), 1 b0).

Preprocessing

Preprocessing software

Freesurfer v6/FSL v6: First, T1 data were processed by the FreeSurfer's crosssectional pipeline (recon-all) which includes motion correction, skull stripping, normalization, intensity correction, volumentric segmentation, and cortical surface reconstruction. Second, the longitudinal pipeline was applied in order to create a robust, unbiased which-subject template

	using robust, inverse consistent registration which increases reliability and statistical power, for the detection of brain structural changes that may occur with intervention. DTI data preprocessing included eddy current and head motion correction using an automated affine registration algorithm. A diffusion tensor model was fitted to the motion-corrected DTI data at each voxel to create individual 3-dimensional FA and MD maps. FSL's BEDPOSTX was used to calculate the distribution of fiber orientations at each brain voxel. Probabilistic fiber tracking was conducted with PROBTRACKX2 implemented in FSL; this method repeatedly samples the distribution at each voxel to produce 'streamlines' that connect voxels from selected seed regions. The following parameters were applied: 5000 streamline samples, 0.5 mm step length, curvature threshold = 0.2. CONN v21: Data preprocessing consisted of functional realignment, slice-time correction, structural segmentation and normalization to the Montreal Neurological Institute (MNI) template, functional segmentation and normalization, and smoothing using a 6-mm Gaussian kernel. Denoising of the blood oxygenation level-dependent (BOLD) signal from physiological and other sources of noise was performed using the CompCor method. The residual BOLD time series were then high pass filtered at 0.01 Hz. Intermediate motion thresholds (0.9 mm slice-to-slice movement and global mean signal below 5 SD) were chosen. Scrubbing was implemented as part of the CONN preprocessing pipeline through the Artifact detection toolbox (ART, http://www.nitrc.org/projects/artifact_detect/) by regressing noise components for outlier scans from the BOLD signal as part of denoising.
Normalization	ROI volumes were extracted from subject-space data. The left middle frontal gyrus from the Harvard-Oxford atlas also used for resting-state fMRI analyses (see below), transformed into individual DTI space, multiplied with diffusion maps and binarized, was used as seed regions for the tractography (in subject-space).<br>Individual T1-weighted images were coregistered to the b0 images, using rigid-body transformation. These registrations were used to transform masks of the left stimulation target to the MD maps.
Normalization template	MNI-152 (for resting-state fMRI)
Noise and artifact removal	See above
Volume censoring	N/A

Statistical modeling & inference

Model type and settings	General linear analyses were modeled with a 2 (groups: anodal, sham) x 2 (time points: pre, post) design (fixed effects, for resting-state fMRI).
Effect(s) tested	The interaction between group and time point was assessed to examine whether functional connectivity alterations from pre to post differed between anodal and sham groups. Age and sex were included as covariates.

Specify type of analysis: ☐ Whole brain ☐ ROI-based ☒ Both

Anatomical location(s): The left middle frontal gyrus from the Harvard-Oxford atlas.

Statistic type for inference (See Eklund et al. 2016)	At cluster-level with height threshold of uncorrected $p < 0.001$ (for resting-state fMRI)
Correction	FDR two-tailed with $p<0.05$ (for resting-state fMRI)

Models & analysis

n/a	Involved in the study
☐	☒ Functional and/or effective connectivity
☒	☐ Graph analysis
☒	☐ Multivariate modeling or predictive analysis

Functional and/or effective connectivity	Pearson correlation of BOLD time-series between seed and resultant cluster regions.
